# From classroom to clinic: innovating radiotherapy treatment planning education through real-world end-to-end case study simulation with an anthropomorphic phantom

**DOI:** 10.1186/s12909-025-06695-w

**Published:** 2025-03-31

**Authors:** Ioannis Genitsarios, Robin Jhagra, Clive Warn, Jesrina Ann Xavier

**Affiliations:** 1 Clinical Educator, German Oncology Centre, Limassol, Cyprus; 2 University Academic and Doctoral Researcher, University of the West of England, Bristol, UK; 3 University Academic and Programme Director, University of the West of England, Bristol, UK; 4 University Academic and Post-Doctoral Researcher, Taylor’s University, Subang Jaya, Selangor Malaysia

**Keywords:** Radiotherapy education, Simulation-based learning, Anthropomorphic phantom, Radiotherapy treatment planning, End-to-end case study learning, Healthcare simulation, Pedagogy

## Abstract

**Background:**

The incorporation of simulation-based learning in healthcare education, particularly in radiotherapy, is necessary for enhancing training and professional competencies to serve patient safety and treatment accuracy. This study aimed to incorporate an innovative end-to-end case study methodology, utilizing an anthropomorphic head phantom, into an undergraduate radiotherapy program at a United Kingdom (UK) based university. The objective was to enhance students’ practical learning and theoretical understanding in radiotherapy treatment planning, a field where precision and accuracy are paramount.

**Methods:**

The study began with an exploratory literature review to identify key educational challenges and opportunities in radiotherapy treatment planning. A qualitative approach was employed, using a focus group methodology to gather in-depth insights from subject experts, including educational and clinical professionals involved in undergraduate radiotherapy teaching. The focus group discussions explored the integration of an anthropomorphic head phantom within a simulated, case study-based training framework. This innovative approach combined practical skills development with theoretical learning, promoting active engagement and mirroring real-world clinical scenarios.

**Results:**

Focus group discussions showed favorability towards the end-to-end case study method in simulation-based learning. Participants emphasized evaluating plans through assessments and using supplementary tools like video guides and workbooks to enhance learning. Incorporating the anthropomorphic phantom marked a notable advancement, offering authentic training possibilities in radiotherapy undergraduate education.

**Conclusions:**

The study demonstrates the potential of integrating an end-to-end teaching concept in radiotherapy education. By providing a realistic and comprehensive training experience, the approach can further enhance student engagement and learning outcomes. While real-world testing is pending, this innovative methodology shows promise in shaping proficient future radiotherapy graduates, highlighting the need for continuous evolution in educational strategies to meet the demands of modern healthcare training.

**Supplementary Information:**

The online version contains supplementary material available at 10.1186/s12909-025-06695-w.

## Introduction

Simulation-based learning (SBL) has become a cornerstone in healthcare education, widely applied across disciplines such as surgery, anesthesia, nursing, and emergency medicine [1–3]. This approach, essential for patient safety and professional competency [4], is supported by the World Health Organization [5] and championed by the UK’s former Chief Medical Officer, Sir Liam Donaldson [6]. In radiotherapy (RT), SBL has facilitated advancements in computerized treatment planning systems [7] and is reinforced by bodies like the National Radiotherapy Advisory Group [8]. The Health and Care Professions Council, UK (HCPC), underscores its value in supplementing clinical placements and aligning practice-based learning with professional standards [9, 10].

Since 2009, healthcare simulation based learning has evolved with platforms like Virtual Environment Radiotherapy Training system (VERT^®^) [11] and Treatment Planning Systems (TPS) such as Eclipse^®^ and RayStation™ [12, 13]. These tools expand training resources for practitioners [14–17] and address limitations in clinical placements, such as capacity constraints [18]. SBL optimizes curriculum design in radiotherapy and oncology education [19–21], enhancing the understanding of radiotherapy’s intricate planning and delivery processes [17].

In radiotherapy education, SBL employs methods such as hands-on training with anthropomorphic phantoms and software simulations to teach treatment planning and delivery. SBL integrates diverse approaches, combining conceptual and practical learning [22]. This integration is further strengthened by radiotherapy’s inherently multidisciplinary nature, with SBL nurturing learner confidence in communicating radiotherapy intricacies across diverse professional groups, including Therapeutic Radiographers (TRs), oncologists, physicists, and allied health professionals [23–25].

While SBL is applicable to both undergraduate and postgraduate education, a significant challenge in undergraduate RT education lies in delivering effective training within constrained timetables and limited clinical placements. Most programs include only one dedicated radiotherapy planning module, hindering the development of critical clinical skills and affecting student confidence and engagement. To address these gaps, this study integrates a fully customizable anthropomorphic phantom into a structured SBL framework, bridging theoretical knowledge with practical application. This approach enhances students’ preparation for clinical practice, beginning with basic simulated training in Year 1 and advancing to hands-on planning in Year 2. The second-year radiotherapy planning module, delivered in Semester 1, is the focus of this study.

The research investigates how integrating an innovative end-to-end case study methodology, utilizing an anthropomorphic head phantom enhances practical learning and theoretical understanding in undergraduate radiotherapy education. Through focus group discussions with education experts, the study evaluates its alignment with curriculum objectives and logistical feasibility. By incorporating this methodology into the undergraduate RT curriculum at a UK university, the research aims to advance pedagogical practices and prepare students for the complexities of modern cancer care [26].

## Context

**Fig. 1:**
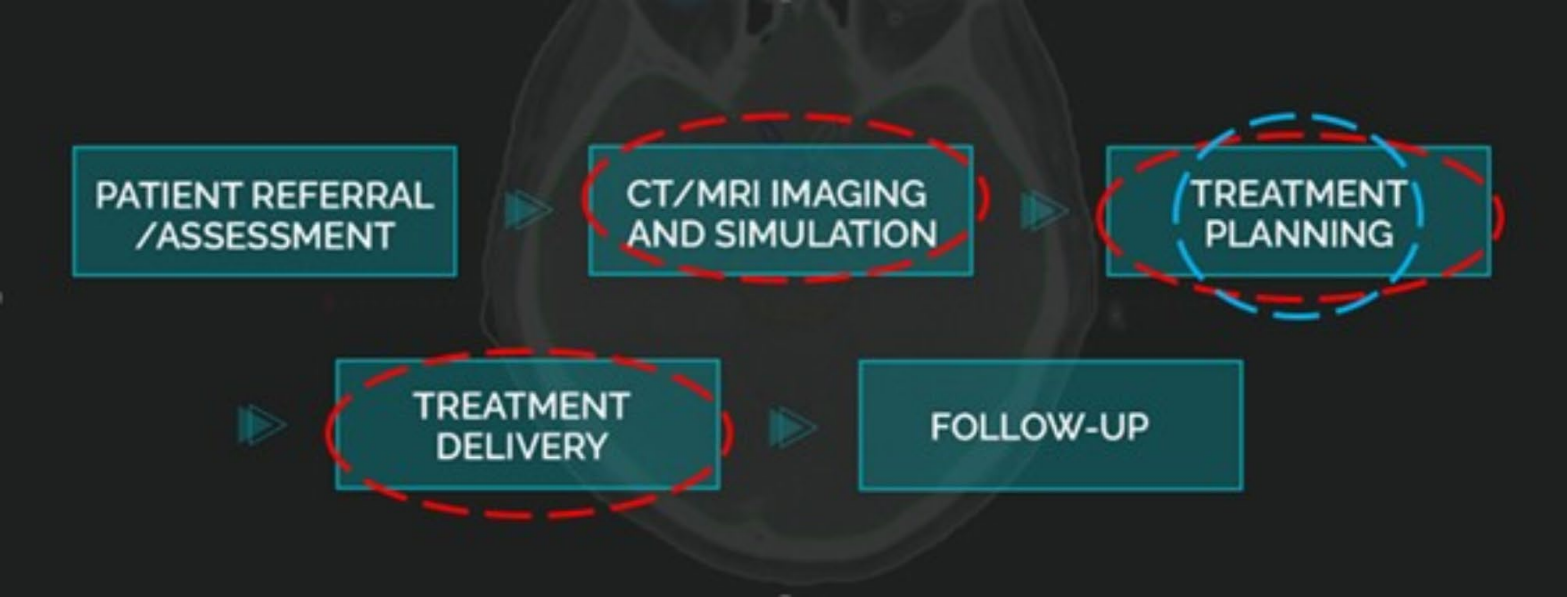
Illustration of radiotherapy patient pathway (Adopted from: MVision™ [27])

For context of this study, the importance of the radiotherapy patient pathway is denoted by the blue circle in Fig. 1. Additionally, the red circled points within the pathway are also relevant to this study, as they represent the overarching process of the radiotherapy pathway.

- Aspects of the pathway requiring TR intervention.
- Aspect of the pathway necessitating simulation training in HEI’s radiotherapy and oncology course.

Fulfilling professional responsibilities in the specified area(s) of Fig. 1 demands robust motor skills, technical knowledge, interprofessional skills, and effective communication with both staff and patients [28]. Simulation replicates these elements, involving a pre-treatment CT imaging process to position the body accurately, often using immobilization devices. Subsequently, datasets are transferred to a TPS, where radiation is prescribed, and beams are mapped and calculated on the tumour to create a treatment plan for patient administration. The approved plan is then transferred to the linear accelerator for treatment delivery. Therefore, understanding RT treatment planning steps through a simulation route is crucial in undergraduate RT training, ensuring trainee practitioners are well-equipped for their forthcoming complex, evolving, and high-stakes nature of the field, contributing to safe, effective, and high-quality patient care [29].

The radiotherapy patient pathway directly influences the proposed methodology by identifying the critical stages where simulation can be integrated to enhance learning. Specifically, the stages of pre-treatment imaging and immobilization, data transfer and treatment planning, are simulated using an anthropomorphic head phantom and the Eclipse^®^ TPS. This approach ensures that students gain hands-on experience in these key steps, thereby improving their practical skills and theoretical understanding in radiotherapy treatment planning.

Contextually, this study examines a HEI that has an established simulation learning capability for undergraduate radiotherapy students. However, the optimal utilization of their current simulation resources, including the Eclipse^®^ TPS, remains unverified. The previous approach in the *Radiotherapy Planning and Dosimetry* module (RPAD) relied on a procedural workbook method for a prostate Intensity Modulated Radiotherapy (IMRT) step-and-shoot case and a tangential breast case scenario. In contrast, this study proposes maintaining a focus on a single IMRT-related treatment site (head) delivered through an integrated and seamless end-to-end case study approach [30, 31]. Although it is possible to incorporate multiple training scenarios using anthropomorphic phantoms designed for different body sites in the current revalidated module, this approach is less favoured due to: (a) the additional costs involved in acquiring custom-designed phantoms and (b) the preference for a single scenario to maintain simplicity and consistency.

### Module revalidation

The transition from the former module, RPAD, to the new revalidated *Principles of Radiotherapy Planning & Simulation (PRPS)* module reflects a weighty milestone in the four-year curriculum revalidation process overseen by the professional body, the HCPC. As a compulsory component of radiotherapy education in the UK, the PRPS module had undergone peer reviewed changes to better align with evolving educational priorities and professional requirements.

**Fig. 2:**
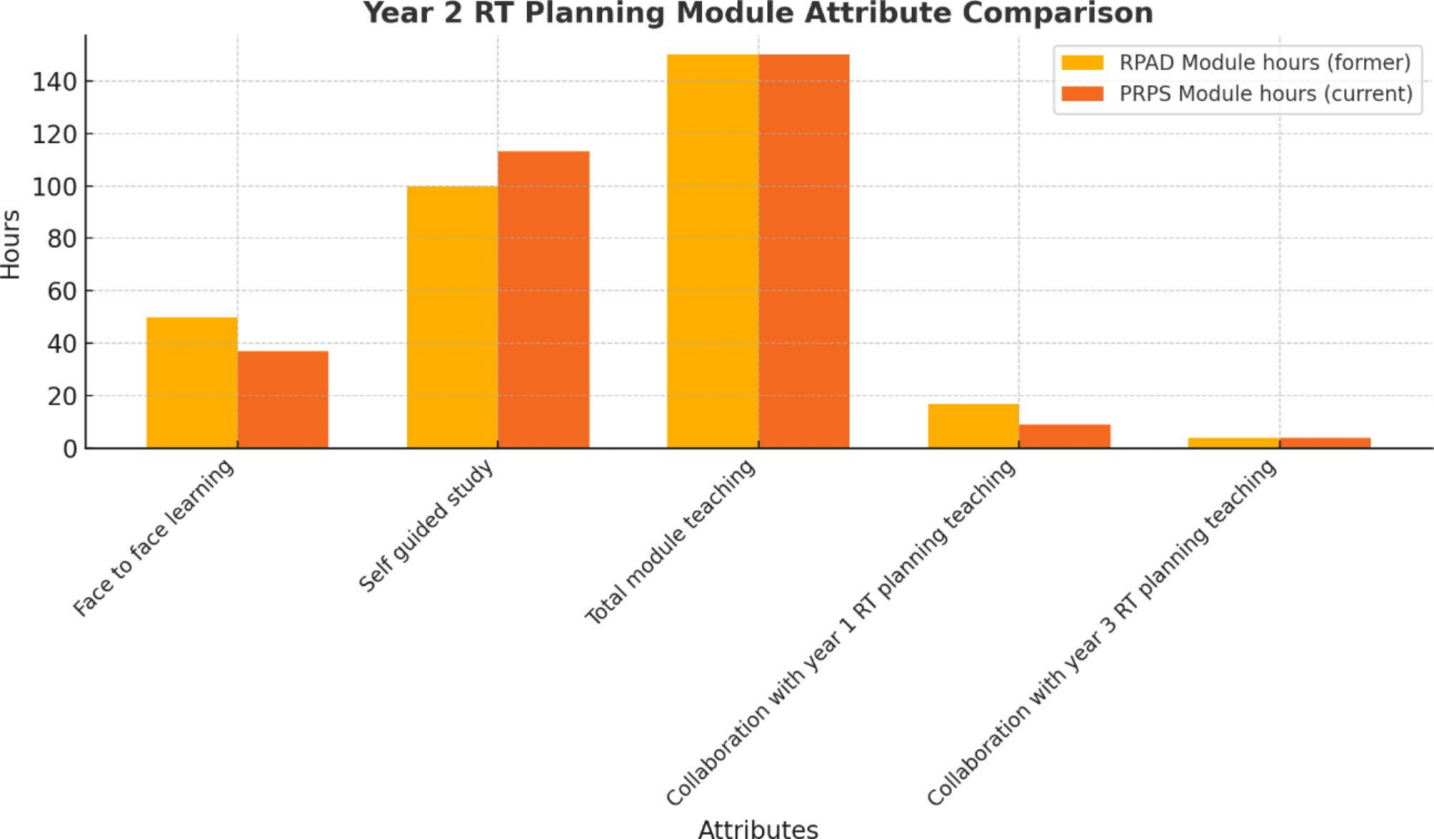
RT planning module attribute comparison

A notable adjustment is the reduction in face-to-face teaching hours alongside an increase in self-directed learning hours Fig. 2. This shift is driven by several key factors. First, the need to adopt greater independent learning aligns with the constructivist principles underpinning modern healthcare education, encouraging students to actively engage with material and take ownership of their learning. Second, the growing availability resources at the university to use the Eclipse^®^ TPS remotely provides opportunities for students to gain experiential learning outside the classroom, complementing face to face instruction. Third, this adjustment addresses the logistical challenges and resources (staff availability) of accommodating larger cohorts.

Figure 2 illustrates that, while the total teaching hours remain consistent between the former and current modules, the importance of optimising face-to-face instruction has increased. This is particularly essential given the slight reduction in dedicated hours for the current module and the marginally decreased time allocated for collaboration with Year 1 planning instruction.

In the pursuit of crafting and evaluating a training proposal that incorporates an anthropomorphic phantom within a simulated, case study-based end-to-end training methodology as outlined in the stages in Table 1, an initiative was embedded within an undergraduate radiotherapy program. This initiative, detailed in this paper, seeks to enrich both practical learning and theoretical comprehension.

**Table 1:** Illustration of vital equipment at key stages in the patient’s radiotherapy process and their functions

Equipment	Purpose	Pseudopatient^®^ head phantom integration
CT Scanner (Simulator)	A scanning device used to mimic the clinical procedure to localize tumour	The phantom can be immobilized and positioned on the scanner safely and accurately
Treatment Planning System (TPS) (Eclipse^®^)	Scans obtained from CT scanner shown on screen where software is utilised by TR to map & position radiation beams virtually	The scanned phantom CT head data is aligned to the real patient CT scan data and the position of the tumour is confirmed
Virtual Environment Radiotherapy Training (VERT^®^) platform	The mapped scan is obtained from the TPS to project onto a large classroom screen for the purpose of student evaluation	The planned data of a student is transferred to (VERT^®^ where the application of different treatment techniques is evaluated by the class

## Learning theories in action

Active learning, which enhances critical thinking, problem-solving skills, and the application of theoretical knowledge to practical situations, has a profound impact on knowledge absorption in radiotherapy education [32]. When students engage physically with learning materials, they deepen their understanding of radiotherapy planning processes and enhance their preparedness for clinical practice. This kinesthetic element is rooted in experiential education, where hands-on experiences activate motor skills, muscle memory, and tactile engagement with complex procedures [33]. By integrating an anthropomorphic phantom into training, learners can simulate patient positioning, scanning, and treatment planning aligning seamlessly with kinesthetic learning theories and promoting a richer grasp of the technical proficiencies required in radiotherapy.

However, immersive kinesthetic learning alone does not guarantee that students will discern all critical features underlying radiotherapy planning. Variation Theory adds a structured dimension by emphasizing the need to experience different facets of a phenomenon, thus enabling learners to pinpoint essential variables and deepen their conceptual understanding [34]. For instance, presenting various tumour sizes, shapes, and locations compels students to adapt their decision-making processes while reinforcing core principles of radiotherapy. This cyclical exposure to novel challenges systematically strengthens adaptability and problem-solving skills - both critical for clinical practice.

Although Howard Gardner’s Multiple Intelligences Theory suggests catering to diverse cognitive abilities (e.g., visual-spatial, bodily-kinesthetic, logical-mathematical) [35], critics argue that it oversimplifies and fragments cognitive capacities [36–38]. In contrast, Variation Theory is deemed superior by some theorists, owing to its robust empirical foundation and nuanced focus on context-driven skill development [39]. In radiotherapy, where technical intricacies are substantial and patient-specific factors vary considerably, Variation Theory’s emphasis on discerning critical features [34, 40] aligns more closely with the domain’s specialized demands.

### Unifying theories through constructivism

Constructivist Learning Theory proposes that learners actively construct knowledge through experiences and reflection, an epistemological stance advanced by figures such as Piaget and Vygotsky [41, 42]. Knowledge is regarded as context-dependent and subjective, shaped by social and environmental interactions. SBL, which immerses students in realistic scenarios that mirror clinical tasks, reinforces these constructivist principles by facilitating learning through active, meaningful engagement. This approach resonates with Vygotsky’s proposition that learning is inherently social and contextually grounded [42].

The integration of an anthropomorphic (humanlike) head phantom within an end-to-end case study framework exemplifies a constructivist approach to radiotherapy education. This approach shifts students from passive observation to active engagement, allowing them to physically position the phantom, perform scans, and plan treatments. By engaging in hands-on practice, students construct their understanding of radiotherapy planning through direct interaction with each stage of the radiotherapy workflow, from patient positioning to treatment planning and delivery [34]. This experiential method not only reinforces theoretical knowledge but also enhances practical proficiency.

Simultaneously, Variation Theory complements constructivism by introducing systematic variation within the learning process. Through exposure to multiple scenarios such as different tumour characteristics, immobilization techniques, and scanning protocols, students gain deeper insights into the principles underlying radiotherapy. This systematic variation encourages learners to generalize their skills across diverse clinical contexts, thereby promoting a robust understanding of both conceptual and practical elements [35]. By integrating these pedagogical approaches, educators maintain authenticity and cater to multiple learning styles, ultimately fostering a comprehensive and adaptive skill set in future radiotherapy practitioners.

Gardner’s Multiple Intelligences Theory supports the concept of differentiated instruction, suggesting that learners benefit from diverse teaching methods, visual aids for visual-spatial learners, hands-on manipulation for kinesthetic learners, and analytical tasks for logical-mathematical learners [35]. Although empirical validation for multiple, distinctly independent intelligences remains limited [36–38], such varied instructional strategies can still be advantageous when integrated into a constructivist framework. In radiotherapy education, where achieving technical accuracy, critical thinking, and collaborative competence is essential, these multi-modal strategies cater to individual learning preferences while maintaining a coherent focus on clinical and procedural mastery.

## Methods

In this research, a custom-crafted anthropomorphic head phantom (Pseudopatient^®^) developed by RTsafe™ (registered at: Regus, Dublin 4 Republic of Ireland), was employed due to its distinctive attributes. Notably, it can be constructed based on actual human CT datasets and is capable of conducting end-to-end pre-treatment verification for intracranial radiotherapy [43]. The hosting university provided a real anonymized patient CT and MRI dataset to ensure the compatibility of imaging files with the RT planning software (Eclipse^®^ v15.7) and VERT^®^ simulation system (Table 1).

**Fig. 3:**
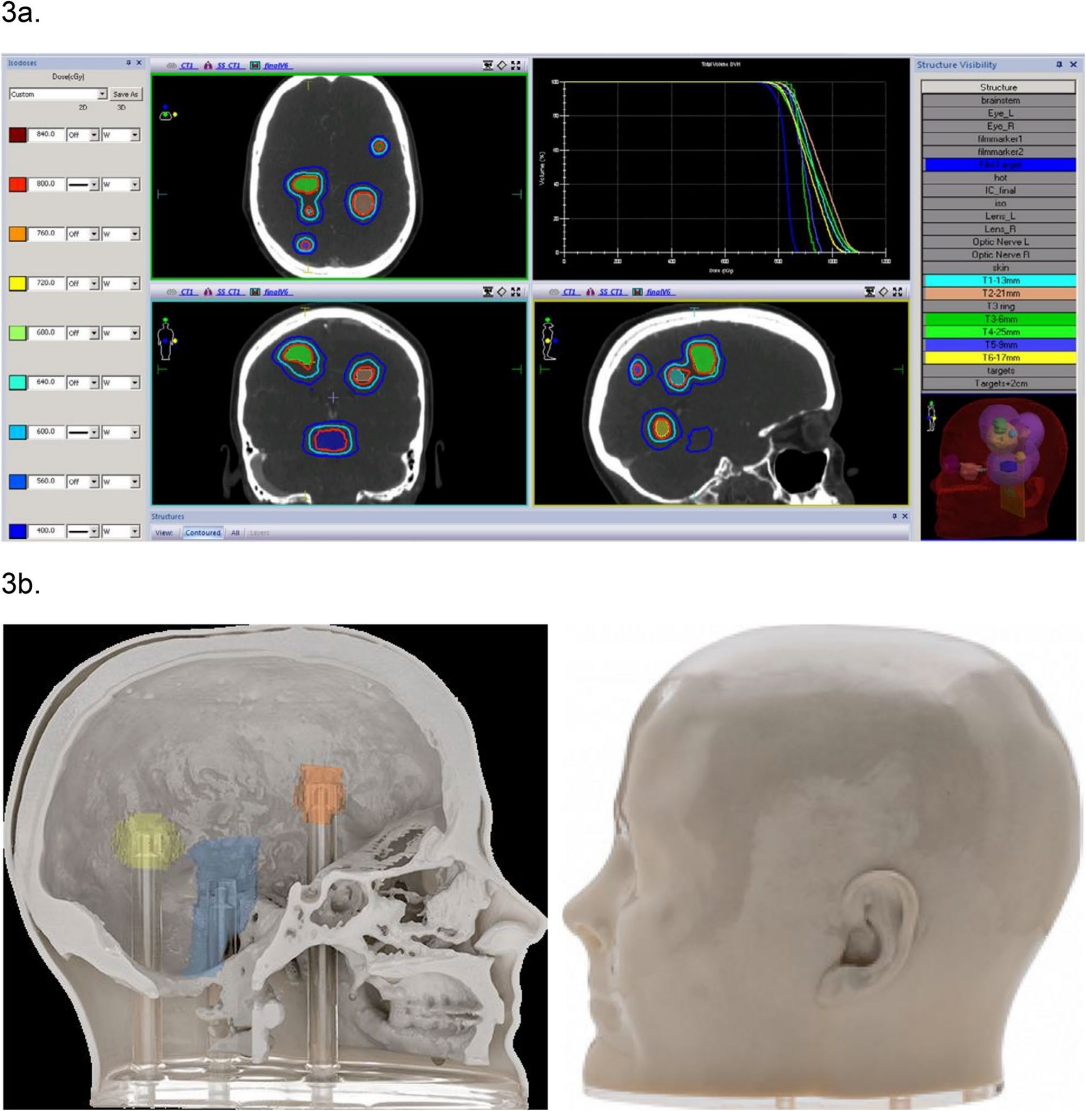
**a** Head phantom imported data in TPS. (Adopted from RTsafe [45]). **b**. Left: Sagittal Section of Pseudopatient^®^ with Targeted Radiotherapy Regions. Right: Lateral View of Pseudopatient^®^ [45]. Note: Sagittal section with color-coded regions indicating areas targeted in radiotherapy or neurosurgical planning, where dosimetric measurements can be obtained

The anthropomorphic phantom integrates materials that replicate both bone and soft tissue equivalence, ensuring contrast in magnetic resonance (MR) and computed tomography (CT) imaging. This feature is important for achieving precision in simulation and planning processes and is particularly significant for verifying dose delivery accuracy and ensuring the safety and efficacy of treatments (Fig. 3a and b) [44]. The phantom’s ability to conduct thorough assessments of spatial accuracy in complex treatments further underscores its value, not only in university settings but also in clinical evaluations.

CT simulation and treatment delivery are introduced in an earlier module at year 1, covering foundational skills such as patient positioning, immobilization, and imaging protocols. These concepts are revisited in the *PRPS* module, where students apply foundational knowledge to advanced planning and simulation using the anthropomorphic phantom. This integration offers hands-on experience, reinforcing understanding and positioning the PRPS module as a critical bridge between introductory and advanced coursework, ensuring a cohesive and progressive learning pathway.

The initial phase involved an exploratory literature review, useful for identifying theoretical underpinnings in SBL and similar pedagogic options. Among various review types a scoping review was deemed most appropriate due to the flexibility and adaptability to new questions and contexts within a confined specialty [46, 47]. It also provided a time sensitive undertaking that could be aligned towards academic semester cycles to aid teaching designs that required diverse sources of knowledge and followed technical trends.

Upon establishing viable simulation teaching methods, the next developmental phase focused on course module design, requiring careful coordination of content and teaching session layout. Emphasizing continuity of learning within the design was key to serving or enabling a positive student experience and engagement [48]. An interpretivist qualitative approach facilitated expert perspectives on the application of this approach within this specific context as it enabled a deep, contextualized understanding of the subjective experiences, complex nuances, and expert knowledge that are integral to this specialized field. As a result, focus groups emerged as the chosen method for qualitative data collection. The literature recognizes the value of a qualitative approach for a comprehensive understanding of experiential research issues [49]. In this context, involving academics and clinical stakeholders familiar with the radiotherapy and oncology programme module specifications was deemed important (Table 2). To facilitate this, a focus group methodology was employed. Participants logged into a planned online session via Microsoft Teams, providing implied consent and engaging in the discussion. The semi-structured approach of the focus group balanced both structure and freedom, allowing deep deliberations. This was essential to obtain valuable insights related to radiotherapy planning within the module constraints and alignment with the overall degree outcomes.

The following stakeholders (educational experts and clinical experts) were recruited in the focus group:

**Table 2:** Participants

Participant 1	Lecturer & Medical Physicist
Participant 2	Programme leader
Participant 3	Lecturer associated with module teaching
Participant 4	Lecturer & clinical scientist (co-responsible for development for educational content, namely workbooks, for some module sessions.
Participant 5	Lecturer & Clinical Scientist (co-responsible for development for educational content, namely workbooks, for some module sessions.

**(** ***N*** **= 5)**

In the process of selecting participants for our study on radiotherapy planning, we strictly adhered to a set of inclusion and exclusion criteria. We included academics and HEI educators who specialize in radiotherapy planning, along with clinical staff such as dosimetrists, clinical scientists, or medical physicists, due to their expertise in planning real cancer cases. We deemed it pertinent to include the programme leader for their comprehensive understanding of the BSc Radiotherapy & Oncology programme, while certain individuals were excluded to maintain research integrity. The module leader for the ‘Principles of Radiotherapy Planning & Dosimetry’ module was omitted to avoid conflicts of interest due to their advisory role in the research. Similarly, the manufacturer of the anthropomorphic phantom and educators or clinicians not directly involved in the subject area were excluded to prevent potential biases. Logistical challenges also influenced the decision to exclude staff from other HEIs, ensuring a focused and manageable virtual meeting. These selection criteria were pertinent for shaping the focus group composition.

The study followed ethical guidelines set by the hosting HEI and was categorized as low risk [50]. The hosting HEI’s research policies were consistently followed, encompassing data management, protection, and destruction protocols. Additionally, an interview guide was developed for this study to ensure consistency and maintain researcher control throughout the data collection process. Before the interview, a comprehensive information sheet detailing the discussion’s nature was distributed to participants to ensure their awareness and comfort with the focus group topics. Participants willingly joined the scheduled online session, and their active presence was construed as implicit consent for participation. Additionally, participants were given another chance to opt out at the session’s outset, demonstrating the researcher’s commitment to respecting participants’ autonomy. The questions were non-sensitive, primarily centering on pedagogic development.

Purposeful sampling, as detailed in Table 2, was employed for its strategic benefits in participant selection [51]. This method facilitated the collection of richer data, more efficient resource utilization, and enhanced the generalizability of the qualitative research findings, which was instrumental for this study. The dialogic data from the focus group interview was recorded utilizing the annotation feature available on Microsoft Teams, facilitating an automated transcription into written language. Excerpts were manually de-identified, assigning participants the labels 1, 2, 3, etc. The related data was securely stored on a OneDrive within the HEI’s IT space in accordance with HEI GDPR compliance regulations.

A thematic analysis approach was carefully employed to delve into a diverse spectrum of topics. The recognition and labelling of data patterns facilitated the grouping of codes into themes (refer to Table 3), relying on patterns and relationships within the educational context, and these inferences were consistent across all researchers involved in the study. Utilizing open-ended questions as a primary instrument, this method was chosen for its inherent ability to uncover nuanced and multifaceted insights that might elude more rigid and structured analysis frameworks. The open-ended nature of the questions allowed participants to express their perspectives freely, contributing to a richer and more comprehensive exploration of the subject matter [52, 53].

## Results

In our findings, we observe a familiarity with the structure of the discontinued undergraduate radiotherapy planning module. This understanding is essential for contextual comprehension, especially considering its discontinuation as part of the program’s revalidation process. The discontinued module served as a solid foundation upon which to build and progress into the new revalidated module cycle, the PRPS module. HEIs conduct revalidation every four years, subject to scrutiny by the UK’s healthcare regulatory body (HCPC) and the professional college (Society & College of Radiographers) to ensure alignment with internal quality assurance and enhancement procedures. The decision to introduce a new end-to-end learning concept using an anthropomorphic phantom arose from the necessity to adapt to the evolving teaching landscape, focusing on the specialized area of radiotherapy planning. This concept holds particular relevance in the second year of the three-year bachelor’s program, where radiotherapy education becomes exclusively pertinent to RT Planning.

**Fig. 4:**
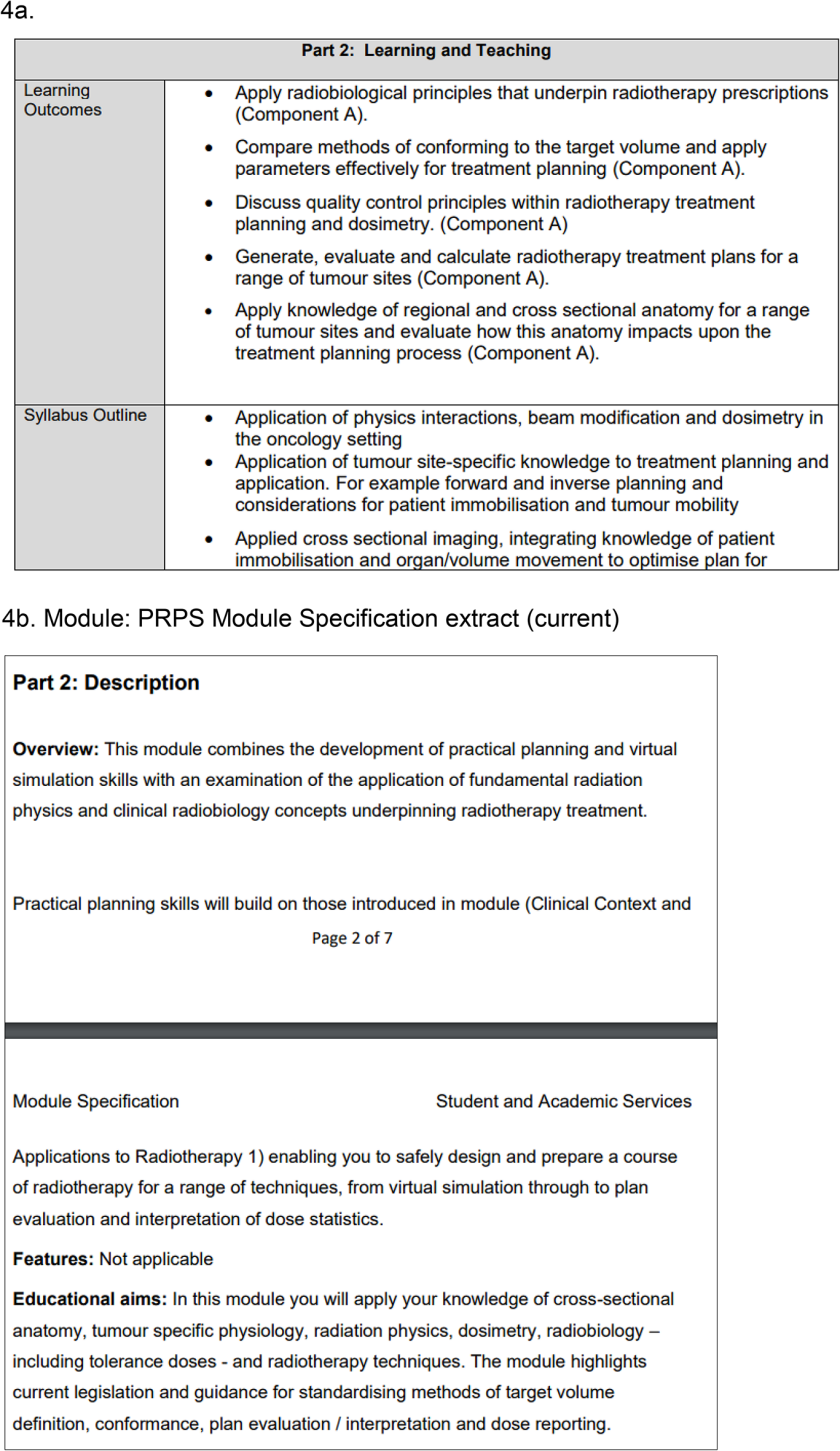
**a** Module: RPAD Module Specification extract (previous). **b** Module: PRPS Module Specification extract (current)

While the proposal garnered support from academic staff within the program, as evidenced by the focus group feedback (refer to Table 4), its inclusion in a new RT planning module ought to be reinforced by insights from a diverse group of education experts. This should include individuals with a combination of academic and clinical expertise who participated in the focus group. Figures 4a and 4b, provide a snippet of relevant curriculum details (previous and current) and fundamentals shared with participants during the focus group, contributing to the overall exploration of the proposed changes in the radiotherapy education curriculum.

An excerpt displaying the schedule and timeline details for the module.

### Revalidated current PRPS module learning outcomes [54]

On successful completion of this module students will achieve the following learning outcomes.

***“MO1****Undertake practical application knowledge of treatment simulation and radiotherapy planning in order to generate*,* calculate and evaluate radiotherapy treatment plans for a range of treatment delivery techniques*,* applying underpinning scientific principles that govern radiotherapy prescriptions*.

***MO2****Discuss and apply international legislation that impacts on quality control principles within radiotherapy treatment simulation*,* planning and dosimetry*.

***MO3****Apply knowledge of regional and cross-sectional anatomy and evaluate how this anatomy impacts upon the treatment planning process*.

***MO4****Critically appraise a range of planning techniques using knowledge gained through enquiry*,* in the classroom setting and the clinical environment*.***”***

Aside from the clinical placement, the ability to teach radiotherapy planning in the final year (year 3) of the program is constrained because of the lack of timetable availability to teach this subject exclusively. Hence the focus of this new teaching methodology was predominantly positioned towards the second year and second term of the program. The focus group questions were tailored to elicit the following information:

- The group’s grasp of the potential enhancement in scenario-based learning through the anthropomorphic phantom revolves around its practical applications for teaching radiotherapy.
- Opinions on the efficacy of an end-to-end case study approach in scenario-based learning covering aspects like module delivery, debriefing, assessment, and the overall learning journey.
- The appropriateness of implementing an end-to-end SBL using the anthropomorphic phantom at the second year of the BSc Radiotherapy and Oncology programme, specifically the RT planning module, assessing its alignment with the curriculum.
- How the anthropomorphic phantom and its dataset can support to serve the module specifications, particularly in terms of teaching and assessment.
- The practicality of completing the end-to-end case study pathway, considering logistical constraints such as cohort size and the use of a linear accelerator in the final stage of the learning journey.
- Formulating effective strategies for scheduling the curriculum of the newly introduced radiotherapy planning module in the forthcoming academic year (2023–2024) and delineating the necessary development of learning resources. This includes specifying details about workbooks, encompassing their content, and determining the requisite number of instructional sessions.

**Table 3:** Key themes and insights

Key Themes	Insights
Practical Applications and Realism	Participants emphasized the value of incorporating the anthropomorphic phantom to replicate real-life clinical workflows. Simulating patient setup errors, conducting CT scans, and transferring datasets to treatment planning systems were considered significant enhancements to traditional training methods. This approach allows students to experience and address realistic challenges within a controlled environment.
Scaffolding Across the Curriculum	Concerns were raised about the timing of introducing complex simulation tasks. Participants suggested aligning earlier exposure to CT techniques with subsequent modules to build a coherent learning progression.
Assessment Strategies	While the end-to-end process was acknowledged as beneficial, assessments should focus on evaluating students’ ability to create and critique treatment plans rather than requiring them to complete all simulation steps. This ensures fairness and feasibility, given potential logistical constraints.
Resource Implications	The time and resource intensity of implementing comprehensive simulations were highlighted, especially for larger cohorts. Suggestions included using video demonstrations to complement hands-on activities, though direct engagement remained preferable for skill development.
Enhanced Confidence and Placement Readiness	Participants reported that this approach could reduce students’ apprehension around treatment planning by offering a holistic understanding of the process. Familiarity with the workflow was expected to improve performance and confidence during clinical placements.

**Table 4:** Summarized extract from focus group data

Question	Participant 1	Participant 2	Participant 3	Participant 4	Participant 5	Coded Deductions
Q1 Having received context to the Pseudopatient phantom, can you see how this device can aid Simulation Based Learning (SBL) within the radiotherapy programme, at HEI?	Interest in simulating setup errors for practical learning	Accepts end-to-end method’s benefits	Value in having tumour structures for contouring in planning	Agrees with all above	Agrees with all above	End-to-end case study approach favored, SBL favored
Q2a What is your opinion regarding an end-to-end case study approach to create a learning journey of a module in simulated based learning? (Delivery, Debriefing, Assessment, opportunities)	Potential to deliver practicals before theory for first-year students	Concerns about assessment methods	Concerns about resources and staff availability	Agrees with all above	Agrees with all above	Assessment to ensure plans are evaluated, logistical consideration of large cohort
Q2b generated due to Q2a.	Pre- and post-session videos useful as a reminder	-	Supplementary use of video in practical sessions	N/A	N/A	Video based guides preferred as assistive material
Q3 As the core RT planning module (PRPS) sits at year 2 of the programme, would an end-to-end simulation case study method using the phantom serve well at this stage?	N/A (Not relevant)	Depends on competency goals and module structure	Adjustable program for proposed method	N/A	N/A	Year 2 RT planning dedicated module highly relevant
Q4 Please see module specification document. How can using a Pseudopatient phantom and dataset serve the module’s specification including assessment?	N/A	-	Phantom useful for creating diverse plans	Agrees	Deeper understanding through end-to-end process	Techniques to learn: Forward plan, Static plan, VMAT plan, Plan Evaluation
Q5 To complete the end-to-end case study pathway within a learning journey, at the final stage, the phantom is designed to be treated on a linear accelerator. However, given the constraints of teaching such as cohort size, can this be achieved?	-	-	-	-	-	N/A
Q6a How can the new PRPS module schema effectively be timetabled for the upcoming academic year? (2023–2024)	Preparing planning teaching in Year 1 by demonstrating phantom scanning	Changes in year 1 module affect the depth of teaching, Introduction is sufficient	CT hands-on approach for small groups, staff in year 2	N/A	N/A	Phantom introduction to be brought in at year 1, CT hands-on training in year 2
Q6b By using a specific learning resource strategy (such as a workbook approach) how many sessions should be developed and what content should this entail?	N/A	No comment	Supports workbooks, undecided on number and content	Unsure	Too early to comment	Agreement on workbook use for teaching guide

The focus group endorsed the end-to-end case study approach for enhancing realism and confidence in treatment planning. It recommended focusing assessments on plan evaluation to ensure feasibility and fairness. Curriculum scaffolding should provide introductory exposure in Year 1 and advanced hands-on applications in Year 2. Supplementary tools like video guides and workbooks can address resource challenges while maintaining skill development.

## Discussion

### Integration of simulation teaching in radiotherapy planning

As highlighted in the paper’s background, comprehensive training in radiotherapy planning is pivotal within the radiotherapy process. Integration of simulation with supplementary resources like workbooks and videos, as outlined by Christensen et al. [55], offers potential to bridge the knowledge gap in radiotherapy, as noted by Mirestean et al. [56]. Despite possessing advanced resources such as Varian’s Eclipse^®^ TPS, Vert^®^, and a Siemens CT scanner, challenges persist in adopting resource-intensive technology-based educational materials The focus group participant 3 attested the specific challenge by highlighting that:

*“…the phantom allows for realistic training by exposing and scanning it*,* offering a step previously unattainable*,* enhancing the practicality of the training program” –* Participant 3.

Thus, a need arises to amalgamate diverse tools focusing on specific notions required at the program’s year 2, as indicated by focus group findings.

Variation Theory was employed to structure the curriculum with varied scenarios, ensuring that students experience different aspects of radiotherapy planning. This approach may help students discern critical features and develop adaptable problem-solving skills, essential for handling diverse clinical situations. By exposing students to multiple scenarios, it is predicted that the ability to transfer theoretical knowledge to practical applications will be enhanced.

Evaluation of the year 2 RT planning module highlighted its suitability, offering comprehensive coverage of the patient simulation pathway within that program level. This module exclusively concentrates on radiotherapy planning and dosimetry, evolving from traditional techniques like 3D-conformal radiotherapy to encompass advanced practices such as IMRT. This shift, guided by technological advancements and stakeholder input from dosimetrists and physicists, integrates contemporary methods emphasizing step-and-shoot and Volumetric Arc Therapy (VMAT) [57] planning techniques. The introduction of the anthropomorphic phantom facilitates practical training in these sophisticated methods, aligning the module with current radiotherapy practice standards and marking a significant improvement in teaching methodologies.

To support the principle of variation theory, SBL was integrated to provide a practical framework for skill development, allowing students to engage in realistic, hands-on training. The use of the anthropomorphic head phantom seeks to facilitate this immersive learning experience, enabling students to practice and refine their skills in a controlled, simulated environment. This approach not only mirrors real-world practice but also provides immediate feedback, essential for reinforcing knowledge and skills.

**Kinesthetic Learning** was incorporated by involving students in the physical manipulation of the phantom, which reinforced learning through direct, tactile engagement. This method complemented the theoretical instruction, helping students internalize complex procedures through muscle memory and hands-on practice.

### Advantages of anthropomorphic phantoms in skill development

The integration of an end-to-end teaching concept was designed to replicate the complete process of radiotherapy planning as it nurtures an interactive learning environment for students [58, 59], thereby enhancing the practicality and applicability of their learning experience. This approach, grounded in Constructivist Learning Theory, emphasizes active engagement and knowledge construction through practical, hands-on experiences. By simulating the entire radiotherapy planning process, students can build a deeper understanding of each step, reflecting Vygotsky’s ideas of learning through social interaction and context.

**Fig. 5:**
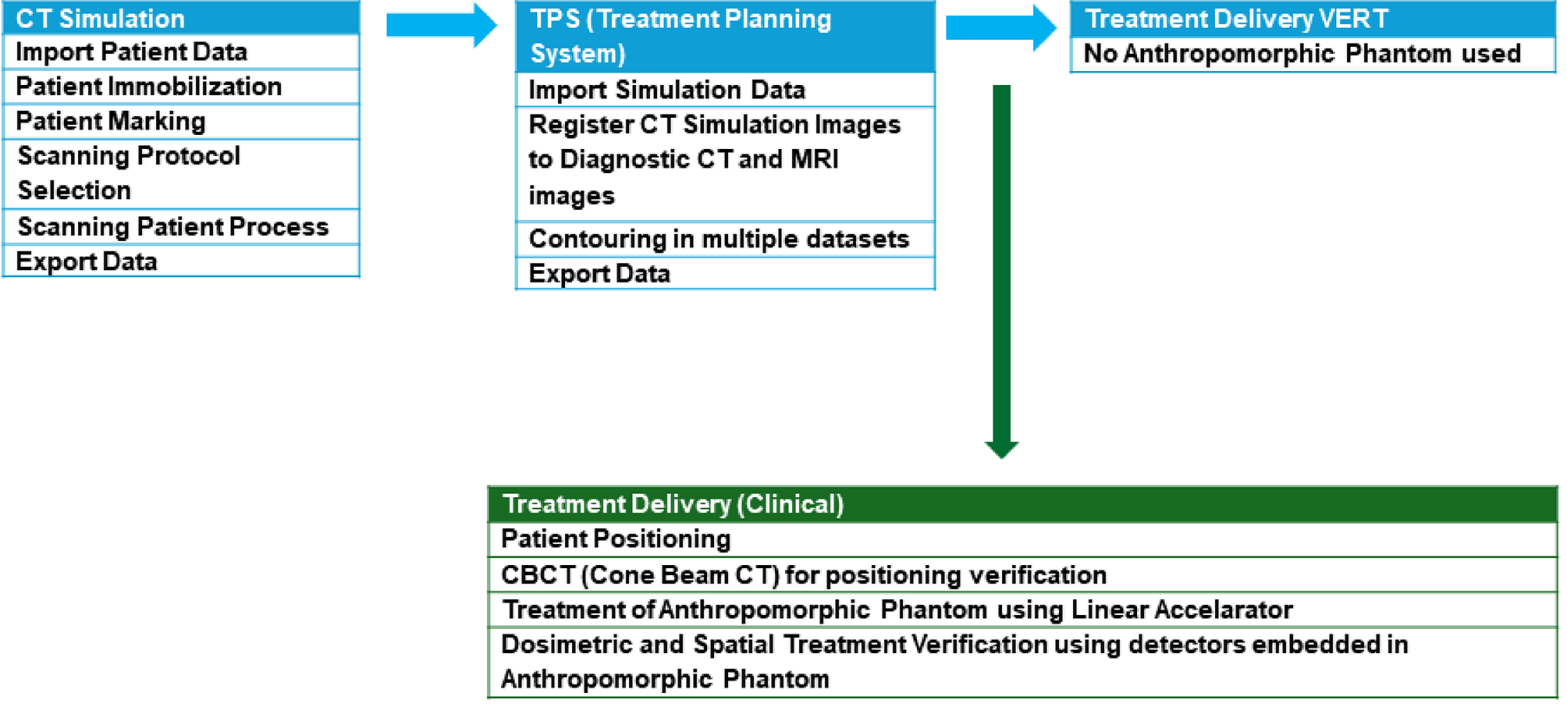
Teaching sessions scheduled on the simulation phase of the radiotherapy patient pathway at the hosting HEI for the new radiotherapy planning module: *Note*: This pathway indicates the use of the phantom and includes the possibility of extending its usage during clinical placement

Additionally, the anthropomorphic phantom was specifically crafted for assuring the quality of clinical procedures in radiotherapy, allowing the validation of treatments before their application in clinical settings. The SBL method supports this by providing realistic, immersive scenarios that mirror real-world practice, enabling students to refine their skills in a controlled environment. Despite the university employing a virtual treatment delivery system, students undergoing clinical placements could replicate their training in a genuine clinical environment, employing the same processes and phantom for practical skill reinforcement (refer to Fig. 5).

Integrating these technologies alongside the phantom’s application required careful consideration of faculty technical skills and administrative clearances. The focus group, recognizing the potential benefits of introducing the phantom into clinical placements, concluded that its implementation might overwhelm current resources and exceed the scope. By applying the principles of Variation Theory, students were immersed in a range of scenarios that highlighted key aspects and differences within clinical contexts. This exposure fostered their ability to identify important details and adjust to varying clinical circumstances, thereby improving their critical thinking and problem-solving abilities—skills vital for proficient radiotherapy planning.

**Fig. 6:**
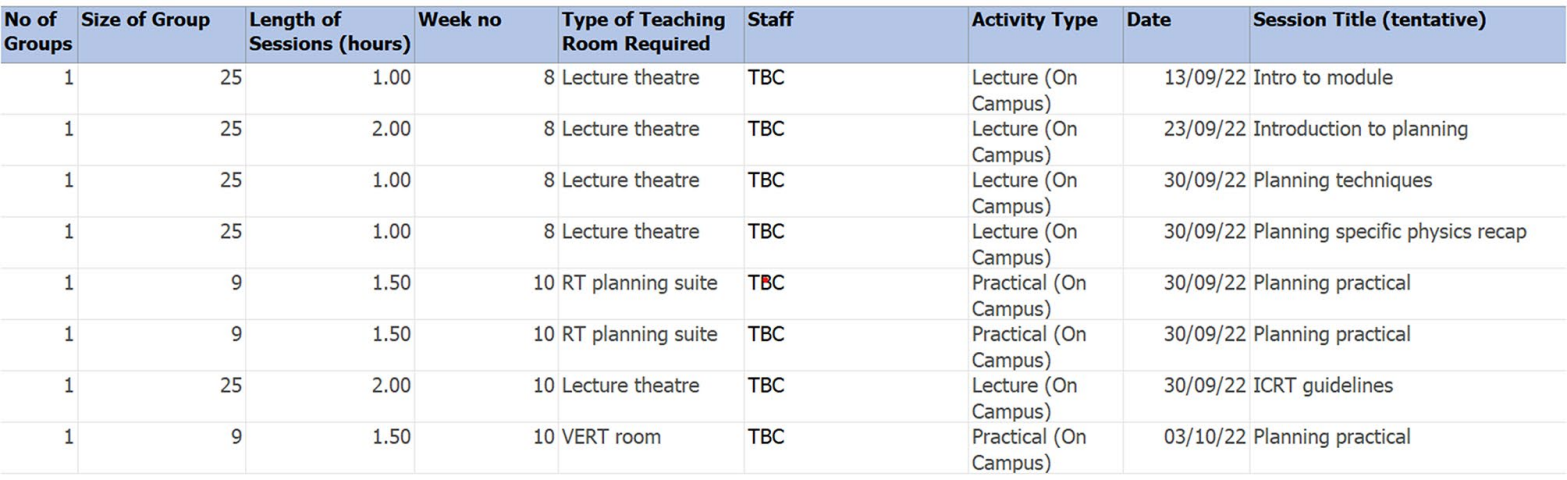
Module Schema template ready to be populated & verified

The formulation of the populated module structure (refer to Fig. 6, within findings) encourages adaptability in educational delivery. Experts from relevant fields contributed to the focus group, setting standards and outlining key competencies, particularly for year 2, aligning with HCPC guidelines [10]. Regarding assessment strategies, they aligned with module specifications, offering flexibility to gather insights on assessment methods from expert focus group members. Educators noted moderate classroom attendance in the prior module run, leading to a proposal to schedule practical sessions that could contribute to the assessment, thereby enhancing student participation. Consequently, students will be required to submit four plans developed during these sessions, and their assessment includes a graded 2500-word evaluative essay. This approach adhered to Gibbs & Simpson’s criteria [60], which emphasizes engaging students in productive learning activities and providing feedback that empowers students to take control of their learning. This initiative aimed to bolster attendance and engagement; an observed need highlighted by educators in the focus group.

By integrating these learning theories, we created a robust educational framework that not only supports theoretical understanding but also enhances practical skills through realistic and varied training scenarios. This comprehensive approach ensures that students are well-prepared for the complexities of radiotherapy planning, aligning their education with current industry standards and best practice.

### Curriculum and timetabling in radiotherapy education

Arranging educational sessions, notably for the recently introduced year 2 RT planning module, demands heuristic approaches, when handling various cohorts and resources [61]. The focus group endorsed incorporating case study-oriented end-to-end simulation teaching within this module, advocating for a structured and unified pedagogical approach. Despite administrative challenges identified by Participant 2 where they expressed that:

“…*timetabling such practical sessions is challenging and will require breaking larger cohorts into smaller groups*,* particularly for CT sessions*,* to ensure effective hands-on learning”* a flexible timetable layout (refer to Fig. 6) was devised to facilitate well-organised scheduling of theoretical and practical sessions.

**Fig. 7:**
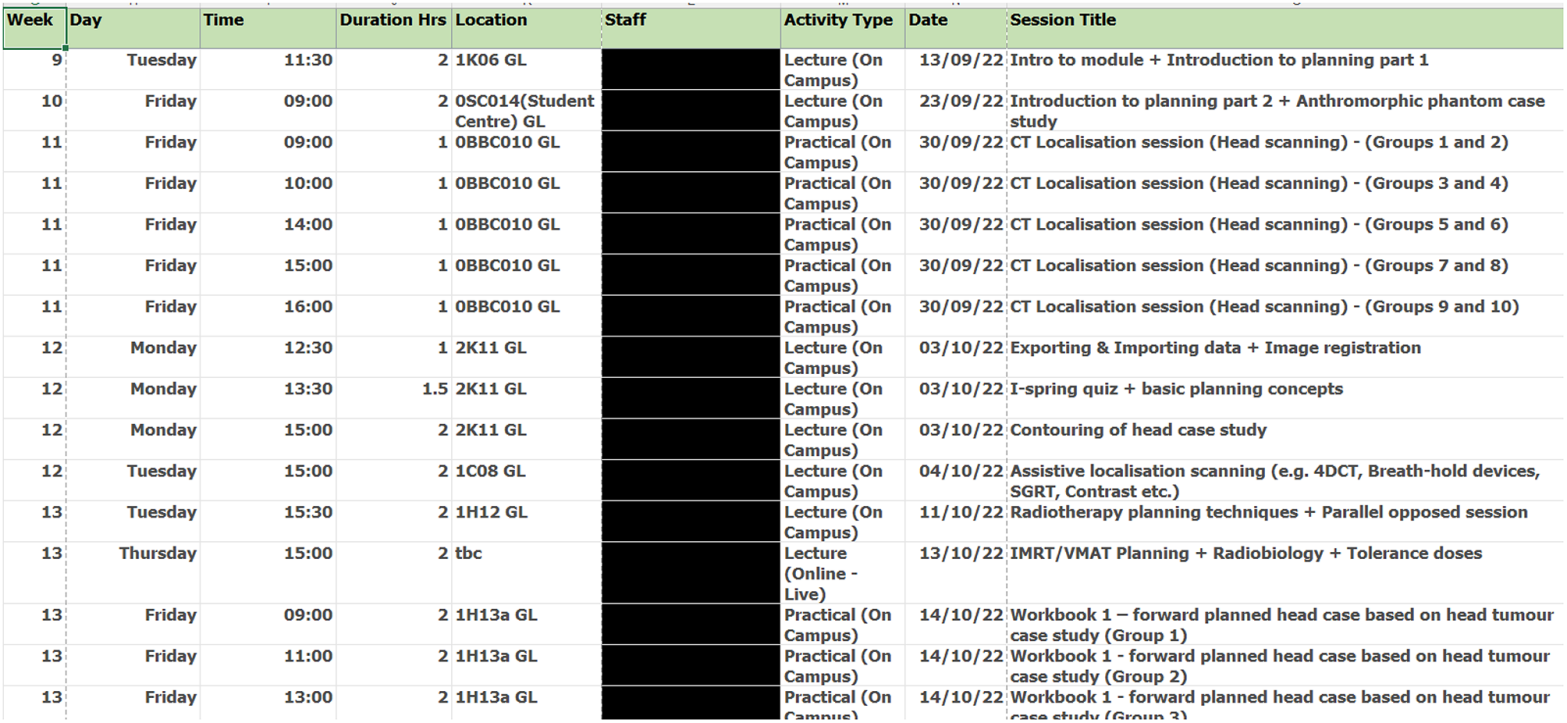
New radiotherapy planning module (PRPS) populated timetable schema

Adjustments were pivotal in managing a considerably large student cohort, particularly by breaking down CT sessions into smaller, more manageable groups. The preliminary populated module structure, as depicted in Fig. 7, showcases the practicality of the final plan. However, the timetable requires alterations to accommodate one-hour CT sessions, catering to the needs of a large group of 50 students. These sessions are set to be bifurcated into two parts within the hour, each part involving five students. One group shall be engaged in a problem-based learning scenario concerning CT scanning and localization, while the other actively participates in setting up the anthropomorphic phantom for subsequent image acquisition.

In the focus group, where experts deliberated, critical topics like step-and-shoot IMRT and VMAT were parsed to ensure an equitable dispersion of subject exposure among all students within the expansive cohort. These techniques are anticipated to persist in the revalidated curriculum unless substantial shifts in clinical practices are observed. While the core focus of dose delivery practices has remained relatively constant, the pandemic-induced landscape has expedited the adoption of hypo-fractionated methods, argued by DiFranko and Birzillo [62] to offer radiobiological advantages to patients. These considerations can be mirrored in the analysis of patient scenarios. The trajectory of radiotherapy appears to be progressing towards ultra-hypofractionation and SRT, as delineated in the ASTRO-ESTRO consensus [63]. These anticipated trends signal a potential evolution in treatment paradigms within the field.

### Methods of teaching delivery

The instruction of radiotherapy planning involves a multimodal teaching approach integrated into workshop sessions as outlined in the module timetable (refer to Fig. 7). This approach includes presentations, handouts, hands-on interactive simulation learning using the Eclipse^®^ TPS, reference guides, and supplementary reading materials. These elements are strategically and creatively combined to enhance subject relevance, foster meaningful engagement, encourage interaction, and facilitate learning in the classroom, ultimately contributing to increased teaching effectiveness [64].

However, to augment student engagement, and learning experiences within workshops, the consensus gravitates towards integrating video-based guides as preferred supplemental resources. The inclusion of videos, alongside workbooks, significantly enriches the learning process and practical skills development in radiotherapy planning [65]. This method not just boosts understanding and accuracy but also enables students to self-evaluate and hone their skills at their own pace, allowing them to replay video clips repeatedly until they have mastered a concept, method, or step within the planning software. Consequently, there is evidence to suggest that this leads to enhanced assessment results and increased confidence levels among students [65].

Mirestean, Iancu [56], underscore the existing knowledge gap in radiation oncology training and accentuate the need for incorporating interactive and innovative educational methods, such as structured teaching modules, to bridge the gap between theoretical and practical knowledge.

Acknowledging the potential of interactive tools, specifically assistive video aids, to enrich the learning experience, a decision was made to defer the development of these interactive elements, including the creation of video aids, to the subsequent academic year. This decision was influenced by imminent constraints related to the module launch and the limited availability of resources for teaching preparation, hence, an established workbook-based platform was chosen as the interim solution to minimise risk of pedagogic disruption.

This choice has proven notably dependable at the hosting HEI, complementing simulation activities and fostering structured learning, as substantiated by Davis et al. [66]. Moreover, supplementing video approach further facilitates self-directed learning, as evidenced in the discontinued year 2 radiotherapy planning module, where workbooks facilitated remote learning opportunities for students to catch up on missed sessions. This multifaceted approach aligns with the varied learning preferences and capacities of students, ensuring a comprehensive and adaptable educational experience.

What we note from this discussion is that the application of an anthropomorphic phantom to education can open up an array of opportunities that enable an enhanced method of learning if it is carefully considered in a curriculum.

### Limitations and future directions

This study, while insightful, is contextually bound to a specific educational setting with a unique anthropomorphic head phantom. Consequently, its applicability to other educational environments, particularly those with differing resources, should be cautiously interpreted.

Our qualitative approach and the specific use of the anthropomorphic phantom in this study may not universally represent radiotherapy educational practices worldwide. This limitation underscores the need for broader methodological applications to validate our findings.

Implementing our end-to-end case study approach may pose challenges in varied settings, particularly where specific equipment and expert personnel are scarce. Institutions aiming to adopt similar strategies should consider these resource constraints.

A key limitation is the potential bias in focus group feedback, as participants, educators and clinicians directly involved in radiotherapy education may have been positively inclined toward the proposed methodology. Another critical limitation is the inherent differences between simulated training and real clinical environments. While the anthropomorphic phantom provides a highly realistic approximation, it cannot replicate the dynamic, unpredictable variables present in clinical practice, such as patient-specific complexities, workflow interruptions, or the emotional and interpersonal aspects of patient care. This gap may limit the direct transferability of skills acquired during the simulation to actual clinical settings. Further research, particularly quantitative studies in varied educational and clinical settings, is needed to evaluate the scalability and efficacy of these methods. As radiotherapy techniques evolve, curricula must adapt dynamically, incorporating learner feedback and emerging educational needs.

The integration of the anthropomorphic phantom into the curriculum is still in its early stages, restricting its application for exporting student-planned cases to a radiotherapy department’s TPS and analyzing geometric and dosimetric data from phantom treatments. Further advancement is required to fully leverage its potential, bridging simulation-based learning with clinical practice through educational innovation.

## Conclusions

The primary objective of this endeavour was to seamlessly integrate an anthropomorphic phantom into the radiotherapy undergraduate program at a higher education institution. Beyond pinpointing an appropriate module for simulation teaching, the ambition was to implement a case study-based approach, employing an end-to-end training method to elevate the overall teaching and learning experiences while nurturing a more engaging atmosphere for students.

By conducting an exploratory review of the literature and engaging in a focused group interview with esteemed experts, we discovered invaluable insights that illuminated practical pathways and solutions, ultimately facilitating the successful integration of the phantom. This collaborative effort not only addressed potential obstacles but also ensured that the integration aligned effortlessly with the curriculum’s pedagogical objectives at the university.

The proposed module structure, shaped by these insights, received approval from the academic team and is expected to be integrated into the curriculum for year 2 undergraduate students. As we strive to implement the outlined recommendations, the utilization of the phantom within an end-to-end teaching and learning methodology, particularly under simulated conditions, will usher in a genuinely immersive approach. This evolution promises to enhance the educational journey for students, creating an environment that truly captivates and engages their learning experience in the next cycle of the academic calendar.

## Electronic supplementary material

Below is the link to the electronic supplementary material.

Supplementary Material 1

## Data Availability

The datasets used and/or analysed during the current study are available from the corresponding author on reasonable request.

## Abbreviations

ASTRO: American Society for Therapeutic Radiology and Oncology
CT: Computer Tomography
CBCT: Cone Beam CT
ESTRO: European Society for Therapeutic Radiology and Oncology
HCPC: Health Care Profession Council
HEI: Higher Education Institute
MRI: Magnetic Resonance Imaging
PRPS: Principles of Radiotherapy Planning and Simulation
SBL: Simulation based Learning
SoR: Society of Radiographers
TPS: Treatment Planning System
VMAT: Volumetric Modulated Arch Therapy
IMRT: Intensity Modulated Radiation Therapy
