# Supplementary material for: From classroom to clinic: innovating radiotherapy treatment planning education through real-world end-to-end case study simulation with an anthropomorphic phantom
Source: BMC Med Educ. 2025 Mar 31;25:465. doi: 10.1186/s12909-025-06695-w (PMC11959824; doi:10.1186/s12909-025-06695-w)
Supplement: Supplementary file 1 — Supplementary Material 1 [file 12909_2025_6695_MOESM1_ESM.docx]

**Semi-structured focus group interview guide**

**Introduction**

**Welcome and Purpose:** Welcome participants and thank them for their time. Briefly explain the purpose of the study, focusing on exploring insights into simulated-based learning (SBL) and assessing the impact of the Head Phantom within the undergraduate RT programme.

**Confidentiality and Voluntary Participation:** Reiterate that the discussion is confidential, participation is voluntary, and responses will be anonymised. Participants may withdraw their data within fifteen calendar days post-interview.

**Consent:** Obtain verbal consent and confirm their understanding of these terms.

**Opening Questions**

Background Information: Can you tell me a little bit about your background and current role as an academic (or clinical practitioner)?

(Aimed at building rapport and understanding the participant’s professional context.)

Experience with Simulated-Based Learning: What is your experience with simulated-based learning (SBL) at university so far?

(To understand the participant’s familiarity with SBL and relevant context.)

**Main Questions**

Q1 Having received context to the Pseudopatient phantom, can you see how this device can aid Simulation Based Learning (SBL) within the radiotherapy programme, at HEI?

Q2a What is your opinion regarding an end-to-end case study approach to create a learning journey of a module in simulated based learning? (Delivery, Debriefing, Assessment, opportunities)

Q2b generated due to Q2a.

Q3 As the core RT planning module (PRPS) sits at year 2 of the programme, would an end-to-end simulation case study method using the phantom serve well at this stage?

Q4 Please see module specification document. How can using a Pseudopatient phantom and dataset serve the module’s specification including assessment?

Q5 To complete the end-to-end case study pathway within a learning journey, at the final stage, the phantom is designed to be treated on a linear accelerator. However, given the constraints of teaching such as cohort size, can this be achieved?

Q6a How can the new PRPS module schema effectively be timetabled for the upcoming academic year? (2023-2024)

Q6b By using a specific learning resource strategy (such as a workbook approach) how many sessions should be developed and what content should this entail?

**Follow-up Probes**

"Could you elaborate on that?"

"Can you give me an example?"

"How did that impact your role/work/practice?"

**Closing Questions**

Additional Comments: Is there anything else you would like to add that we haven’t covered?

Participant Questions: Do you have any questions about this research or how your data will be used?

**Conclusion**

Thank the participant for their time and valuable input. Explain the next steps, including transcription and analysis, and how they can request a copy of the results.
